# Pretreatment Serum Uric Acid as an Efficient Predictor of Prognosis in Men with Laryngeal Squamous Cell Cancer: A Retrospective Cohort Study

**DOI:** 10.1155/2019/1821969

**Published:** 2019-04-16

**Authors:** Chi-Yao Hsueh, Mingxi Shao, Wenjun Cao, Shengjie Li, Liang Zhou

**Affiliations:** ^1^Department of Otolaryngology, Eye & ENT Hospital, Shanghai Medical College, Fudan University, Shanghai, China; ^2^Shanghai Key Clinical Disciplines of Otorhinolaryngology, Shanghai Medical College, Fudan University, Shanghai, China; ^3^Department of Clinical Laboratory, Eye & ENT Hospital, Shanghai Medical College, Fudan University, Shanghai, China

## Abstract

**Purpose:**

Uric acid (UA) is a major antioxidant molecule that has been hypothesized to have a protective effect against cancer-induced oxidative damage. The aim of the present study was to investigate whether preoperative levels of serum UA are associated with the prognosis of laryngeal squamous cell cancer (LSCC).

**Methods:**

A total of 814 male LSCC patients (followed up for five years) and 814 normal control subjects were enrolled from January 2007 to December 2011. The rates of total mortality and cancer mortality were 23.46% and 21.36%, respectively. The prevalence of overall survival (OS), disease-free survival (DFS), and cancer-specific survival (CSS) was analysed using the Kaplan-Meier method. Univariate and multivariate Cox regression models were evaluated to identify UA as a prognostic factor.

**Results:**

The serum UA and UA/Cr (creatinine) ratio levels were significantly reduced (*P* < 0.001 for both) in the LSCC group compared with the control group. The applied multivariate Cox regression model analysis found that low levels of UA and the UA/Cr ratio were independent poor prognostic factors for OS (UA (HR (95% CI) = 1.458 (1.095–1.942)), UA/Cr ratio (HR (95% CI) = 1.337 (1.004–1.780))), DFS (UA (HR (95% CI) = 1.504 (1.131–2.001)), UA/Cr ratio (HR (95% CI) = 1.376 (1.030–1.839))), and CSS (UA (HR (95% CI) = 1.494 (1.109–2.012)), UA/Cr ratio (HR (95% CI) = 1.420 (1.049–1.923))). The patients with high UA (>0.310 mmol/l) and UA/Cr ratio (>3.97) experienced five more years of OS, DFS, and CSS than did patients with low UA (<0.310 mmol/l) and UA/Cr ratio (<3.97) levels.

**Conclusion:**

High preoperative UA serum levels were identified as an independent prognostic factor associated with improved clinical outcomes among LSCC patients.

## 1. Introduction

Laryngeal cancer is one of the most common cancers of the head and neck/respiratory tract, of which the estimated incidence and mortality in China in 2015 were 26,400 and 14,500, respectively [1–3]. The predominant histological type of laryngeal cancer in China is laryngeal squamous cell cancer (LSCC) [4]. Total laryngectomy and chemoradiotherapy have been the primary treatment methods for advanced-stage LSCC (stage III or IV). Unfortunately, conventional fractionated radiotherapy and total laryngectomy are currently not considered optimal strategies for LSCC treatment [5]; they result in complete loss of voice and can cause patients to experience impaired swallowing, which significantly affects the quality of life in terms of nutrition, social functioning, and personal hygiene [6, 7]. Even more unfortunate, the five-year survival rate of LSCC has decreased over the past 40 years from 66% to 63% [8]. To date, several histopathological prognostic factors, such as tumour size, histological subtype or grade, vascular invasion and lymph node metastases, and oxidative stress, have been considered important factors for patients with LSCC [9–12].

Uric acid (UA), the naturally occurring product of purine metabolism, is a major water-soluble antioxidant molecule in human plasma. It has metal-chelating properties as well as the ability to scavenge nitrogen radicals and superoxide in the plasma, which helps block the generation of strong oxidant peroxynitrite [13, 14]. In 1981, Ames et al. first reported that UA may provide an antioxidant defence against oxidant- and radical-caused ageing and cancer in humans [15, 16]. Recently, increasing evidence has shown that higher UA blood levels may indeed be protective and could serve as a prognostic marker for different types of cancer [17–22]. For example, Dziaman et al. [18] reported that colon cancer patients with low UA plasma levels (values lower than 277.8 mM) had shorter survival times than those with high UA levels (OS_60 months_ 40% vs. 66%, respectively; *P* = 0.006), which suggested that UA is an efficient predictor of survival in colon cancer patients; Strasak et al. [23] found that serum UA levels in the highest tertile (>5.8 mg/dl) were associated with a lower risk of mortality from any cancer among males in a large general population-based cohort study. Moreover, Kühn et al. [21] also reported that UA levels were inversely associated with breast cancer risk (HR_Q4 vs. Q1_ (95% CI): 0.72 (0.53, 0.99), *P*_linear trend_ = 0.043) and cancer mortality (HR_Q4 vs. Q1_ (95% CI): 0.75 (0.58, 0.98), *P*_linear trend_ = 0.09) and indicated that higher levels of UA may be an efficient predictor of prognosis in patients with breast cancer. However, contradictory evidence from other studies does not support this hypothesis, suggesting instead that elevated levels of UA may raise the risk of cancer mortality and reduce life expectancy [24–26]. In the field of head and neck cancer, only one study reported that UA levels were significantly elevated in T1-3 nasopharyngeal carcinoma patients [27]. To the best of our knowledge, however, the level of UA in patients with LSCC and the association between UA and the prognosis of LSCC have not been investigated sufficiently.

Although “omics”-based technology has enabled faster identification of their probable disease risk, prognosis, and/or response to treatment biomarkers, the validation of biomarkers is still stymied by high cost and poor output of results. Precision medicine relies on validated biomarkers with which to better classify patients by their probable prognosis [28]. Currently, as a simple, rapid, and reliable parameter, UA is recommended for predicting cancer prognosis. Therefore, we hypothesized that UA has a protective effect against LSCC-induced oxidative damage. In some previous studies, secondary data from health insurance and primary care were used for statistical analyses; however, detailed adjustment for potential confounders was not possible. To meet this need, we conducted a large-sample case-control and cohort study to detect and compare the serum UA levels of LSCC subjects and to investigate the possible association between serum UA levels and the prognosis of LSCC.

## 2. Materials and Methods

### 2.1. Study Population

This study was approved by the Ethics Committee of the Eye & ENT Hospital of Fudan University in Shanghai, China, and was conducted in accordance with the Declaration of Helsinki. Written informed consent for the use of any clinical data for research was obtained for all patients. Patients with LSCC were recruited from the Department of Laryngeal Medicine at the Eye & ENT Hospital of Fudan University using a primary cohort of consecutive patients who underwent partial or total laryngectomy between January 1, 2007, and December 31, 2011, as their first curative treatment option. All patients were followed up by telephone, and outpatient records were obtained every 3 months during the first 2 years and every 6 months thereafter until death to remain up-to-date on patient survival status, disease progress, and time of death. The last follow-up was September 30, 2016. DFS was recorded from the date of laryngectomy to the date of recurrence within the follow-up period. CSS was recorded from the date of surgery until death because of intercurrent disease within the follow-up period. OS was recorded from the date of surgery until death. Normal controls were recruited consecutively from subjects who participated in yearly health screenings during the study period. Written informed consent for the use of any clinical data for research was obtained for all normal controls. Medical examinations were performed by respective physicians for all subjects at the Eye & ENT Hospital of Fudan University.

### 2.2. Inclusion Criteria

**Figure 1:**
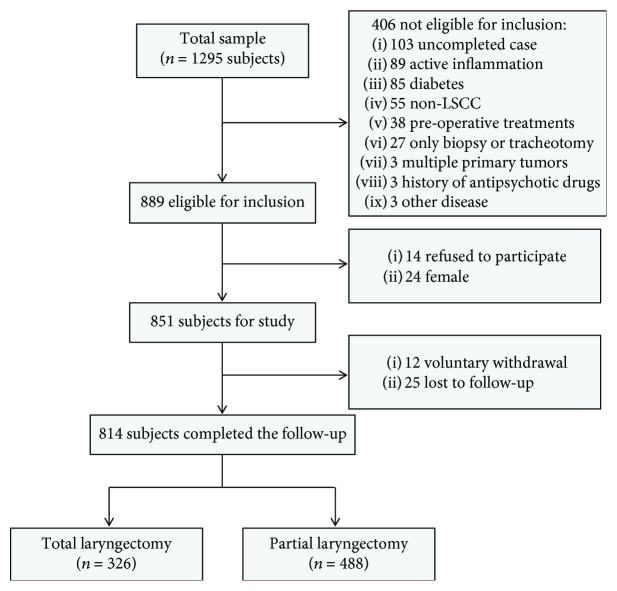
Study cohort flow diagram.

To select the LSCC group, 1295 LSCC patients who visited the Department of Laryngeal Medicine at the Eye & ENT Hospital of Fudan University between January 1, 2007, and December 31, 2011, were enrolled. The study cohort flow diagram is shown in Figure 1. In China, the incidence of LSCC was approximately 20 to 30 times higher in men than in women [2]; as such, female LSCC patients were excluded from this study (only 24 female subjects met the inclusion criteria). A final total of 814 male LSCC subjects were eligible for the study.

A total of 485 normal control subjects were excluded based on the inclusion criteria. The exclusion rate for the control group was 30.69%. We performed propensity score matching analysis (1 : 1) to establish a control group from among the 2000 normal control subjects who met the inclusion criteria. The selected variable was age. A final total of 814 male control subjects were eligible for the study.

The inclusion criteria and selection process for the included LSCC patients were as follows:

1. All patients had histologically proven squamous cell carcinoma, confirmed by pathology and classified under the seventh edition of the TNM-UICC/AJCC stage classification (diagnostic criteria)
2. Patients were a minimum of 18 years old
3. Blood samples were collected before the patients' pretreatment
4. Complete clinical, laboratory, imaging, and follow-up data were collected
5. LSCC subjects were selected from the population of inpatients
6. LSCC subjects voluntarily agreed to participate in this study, provided written informed consent, and were able to understand and comply with the research requirements

The inclusion criteria for the control subjects were as follows:

1. Subjects were a minimum of 18 years of age
2. Subjects were male
3. Subjects had no LSCC
4. Complete clinical and laboratory data were collected
5. Subjects voluntarily agreed to participate in this study, provided written informed consent, and were able to understand and comply with the research requirements

The exclusion criteria for the LSCC patients and control subjects were as follows:

1. Subjects suffered from systemic diseases, such as infectious diseases (infection with hepatitis B and hepatitis C, infection with human immunodeficiency virus, or having a positive result on the screening test for one or more of those infections), autoimmune disease, rapidly progressive visceral disease, and cancer
2. Subjects were taking medications that could influence serum UA levels, for example, diuretic, benzbromarone, and losartan
3. Subjects suffered from kidney disease or had a history of kidney disease
4. Subjects suffered from hyperuricaemia or gout
5. Subjects had undergone surgical intervention or an operation

### 2.3. Data Collection

Clinical and demographic information was obtained from the medical data platform of Eye & ENT Hospital by trained staff using standardized data collection and quality control procedures, which produced reliable data for analysis. For each patient, clinical and demographic information was collected from the medical data platform independently by two independent investigators. Moreover, the two independent investigators consulted the medical data platform again to resolve any disagreements. The data included the following demographic information: age, sex, UA levels, creatinine (Cr) levels, alcohol drinking habits, blood pressure, glucose levels, and smoking habits. The following clinical information was collected as well: medical history, date of diagnosis, tumour subsite, tumour size, local and regional extension category of the primary tumour, clinical stage, differentiation grade, surgical therapy experienced, and level of neck dissection. Follow-up data included date of primary resection, date and type of relapse, date of diagnosis of metastatic disease, and date of death.

### 2.4. Laboratory Assays

Patients' peripheral blood samples were collected at the first time of visits at our hospital. Meanwhile, blood samples were collected before the patients' pretreatment. Blood samples for biochemical measurements were obtained via standard venipuncture of the veins in the antecubital fossae (anterior elbow veins) the morning after subjects had fasted for 8 hours. Detection methods for UA and creatinine levels have been previously described in detail [16]. Briefly, quantification of serum UA and creatinine was measured by enzymatic colorimetry using a commercially available kit (Roche Diagnostics GmbH, Mannheim, Germany). Internal controls were analysed daily over the 10-year period, with typical monthly CVs of 2%–4% and no significant changes in the values.

### 2.5. Statistical Analysis

All analyses were performed using SPSS 13.0 software (SPSS Inc., Chicago, IL). All figures included in this study were created using GraphPad Prism 6 software (La Jolla, CA). Patient demographics and clinical characteristics were displayed as frequency counts and percentages. The UA/Cr ratio equalled UA∗1000/Cr. The data are presented as the mean ± standard deviation (SD). Normality was assessed using the Kolmogorov-Smirnoff test. Independent Student's *t*-test and chi-square test were used to compare the characteristics of subjects between the groups. A one-way ANOVA test was used to compare UA, Cr, and UA/Cr ratio levels among the three groups. Receiver operating characteristic (ROC) analysis was performed to identify the sensitivity and specificity for UA and the UA/Cr ratio and the optimal cut-off values for predicting LSCC. Upon verification of the prediction value of UA and the UA/Cr ratio as a continuous variable, the relevance of the clinical stage of the categorized UA and UA/Cr ratio was evaluated by assigning subjects to two groups based on the optimal cut-off value. Logistic regression analyses were performed to identify the independent risk factors for LSCC patients. Moreover, the associations between UA, Cr, and the UA/Cr ratio and clinical stage of LSCC were examined using multiple linear regressions. Univariate Cox regression analyses were performed to determine the association between UA, UA/Cr ratios, and different clinicopathological parameters on OS, DFS, and CSS. After application of the univariate Cox regression analysis, a multivariate Cox regression analysis (adjusted for covariates) was used to analyse the association between the UA and UA/Cr ratio and the OS, DFS, and CSS of LSCC. The UA level and the UA/Cr ratio had a significance of *P* < 0.05 in the multivariate Cox regression analysis; thus, the regression models for OS, DFS, and CSS curves were plotted using the Kaplan-Meier method. A two-sided *P* < 0.05 was considered statistically significant.

## 3. Results

### 3.1. Patient Characteristics

A total of 814 male LSCC subjects and 814 male normal control subjects were eligible for inclusion in the study. The median follow-up period was 72 months (from a range of 3–116 months), and 623 (76.54%) patients were still living at the time of the last follow-up visit. The median subject survival time was 38 months (range, 3–105 months). The rates of total mortality and cancer mortality were 23.46% (*n* = 191) and 21.36% (*n* = 174), respectively. Based on the TNM stage, the LSCC subjects were categorized into four stages, of which 195 (23.96%) were classified as stage I, 302 (37.10%) as stage II, 216 (26.54%) as stage III, and 101 (12.41%) as stage IV. Following initial entry into this study, 36.61% of the patients (*n* = 298) experienced tumour recurrence, of which the median recurrence interval was 23 months (range, 1–95 months). The baseline characteristics and clinicopathological features of the study subjects are shown in Table 1.

### 3.2. Comparison of UA, the UA/Cr Ratio, and Cr between the LSCC and Control Groups

The UA, UA/Cr ratio, and Cr levels in patients with LSCC are shown in Table S1. The serum UA and UA/Cr ratios were significantly higher (*P* < 0.001) in the control group than in the LSCC group. Logistic regression analysis showed that high levels of UA (OR = 0.671, *P* = 0.002, 95% CI = 0.018-2.165) and the UA/Cr ratio (OR = 0.584, *P* < 0.001, 95% CI = 0.439-0.778) were independent protective factors for the development of LSCC.

### 3.3. ROC Analyses of the Studied Variables

**Figure 2:**
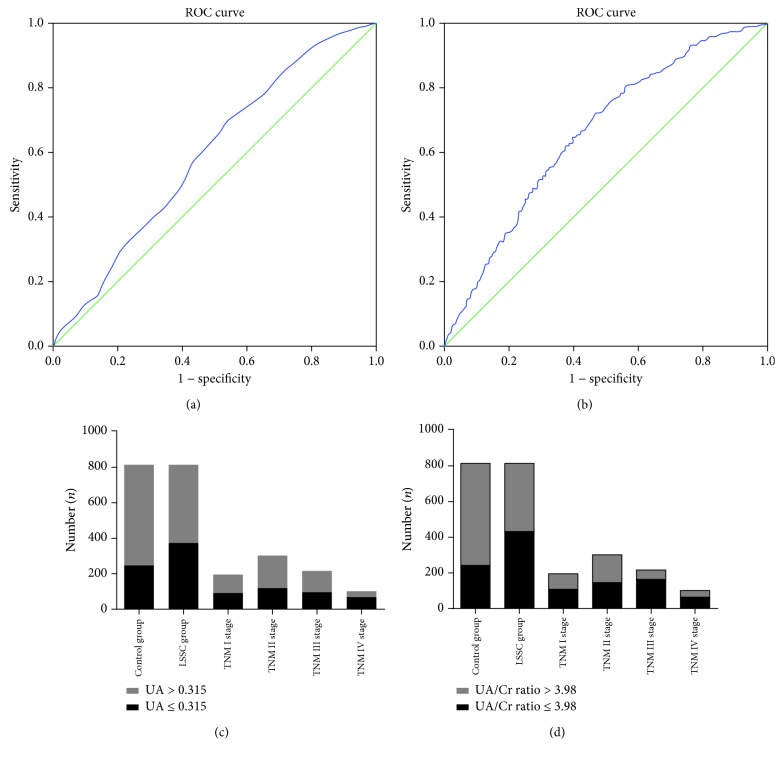
Receiver operating characteristic (ROC) curve analysis for uric acid (UA) (a) and the UA/Cr ratio (b) in predicting laryngeal squamous cell cancer (LSCC). The patients with different LSCC severities (TNM stages) and the control group were stratified by UA (c) and the UA/Cr ratio (d).

Results of the ROC analyses with the studied variables are shown in Figure 2. The AUROC (area under the ROC curve) values for UA and the UA/Cr ratio used to distinguish LSCC patients from control subjects were found to be 0.594 and 0.659, respectively. The best UA cut-off value was 0.315 (*P* < 0.0001, 95% CI = 0.566–0.621), with a sensitivity of 70.27% and a specificity of 45.83% (see Figure 2(a)). The best UA/Cr ratio cut-off value was 3.98 (*P* < 0.0001, 95% CI = 0.632–0.685), with a sensitivity of 72.24% and a specificity of 53.20% (see Figure 2(b)).

### 3.4. Comparison of UA, Cr, and the UA/Cr Ratio in Patients with LSCC, Stratified according to Demographics and Clinical Characteristics

Based on the TNM stage, the mean levels of serum UA and the UA/Cr ratio were the lowest among the stage IV patients, followed by stage III, stage II, and stage I patients (all *P* < 0.05). A similar trend was observed when UA and UA/Cr ratio levels among the four stages were compared with respect to T/N. Details are shown in Table 2.

Multiple linear regression models (adjusted for age, hypertension, smoking, and drinking) for the associations between UA, Cr, and UA/Cr ratios and the overall stage of LSCC were used. The results showed a significant association between UA (*B* = −1.824, *P* < 0.0001, 95% CI = −2.437–-1.002) and the UA/Cr ratio (*B* = −0.079, *P* = 0.045, 95% CI = −0.321–-0.024) in the TNM stage of LSCC. Details are shown in Table 3.

### 3.5. Comparison of Stratified UA and UA/Cr Ratio Levels in Patients with LSCC

LSCC patients were classified into two groups according to the UA cut-off value (UA ≤ 0.315 vs. UA > 0.315) and the UA/Cr ratio (UA/Cr ratio ≤ 3.98 vs. UA/Cr ratio > 3.98) in each subgroup. The distribution of the UA and UA/Cr ratio values differed significantly when the patients were stratified by T/N/TNM classification, and lower levels of UA (≤0.315) and the UA/Cr ratio (≤3.98) were significantly associated with LSCC severity. Details are shown in Table S2, Table S3, and Figures 2(c) and 2(d).

### 3.6. Univariate Cox Regression Analysis for OS, DFS, and CSS in Patients with LSCC

The univariate analysis identified low UA levels (≤0.315 mmol/l versus >0.315 mmol/l, HR = 1.466 (95% CI = 1.104–1.947), *P* = 0.008) and low UA/Cr ratio levels (≤3.98 versus >3.98, HR = 1.308 (95% CI = 0.984–1.737), *P* = 0.044) as poor prognostic factors for OS in this study cohort (see Table 3). Similar results revealed a low UA level and low UA/Cr ratio level as poor prognostic factors for DFS and CSS (see Table 4).

### 3.7. OS, DFS, and CSS Outcomes

**Figure 3:**
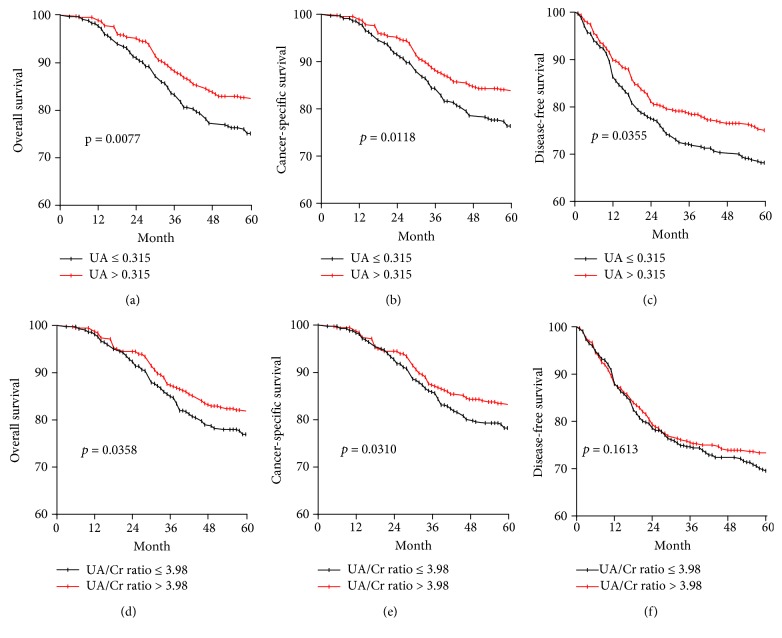
Kaplan-Meier OS, DFS, and CSS curves stratified by the cut-off value in terms of UA and the UA/Cr ratio. (a) OS curves stratified based on the UA category. (b) CSS curves stratified based on the UA category. (c) DFS curves stratified based on the UA category. (d) OS curves stratified based on the UA/Cr ratio category. (e) CSS curves stratified based on the UA/Cr ratio category. (f) DFS curves stratified based on the UA/Cr ratio category.

Patients with UA ≤ 0.315 had poorer 5-year OS (75.1% vs. 82.7%, *P* = 0.0077), DFS (68.2% vs. 75.1%, *P* = 0.0355), and CCS (76.4% vs. 84.1%, *P* = 0.0118), compared with patients with UA > 0.315 (Figures 3(a)–3(c)). Patients with a UA/Cr ratio ≤ 3.98 had poorer 5-year OS (78.2% vs. 82.9%, *P* = 0.0358), DFS (69.6% vs. 73.3%, *P* = 0.1613), and CCS (78.3% vs. 83.5%, *P* = 0.0310), compared with patients with a UA/Cr ratio > 3.98 (Figures 3(d)–3(f)).

### 3.8. Multivariate Cox Regression Analysis for OS, DFS, and CSS in Patients with LSCC

In addition to age, tumour subsite, operation therapy, tumour size, TNM stage, T stage, and N stage, reduced UA level (≤0.315) was a key predictive risk factor for OS (HR = 1.458, 95% CI = 1.095–1.942), DFS (HR = 1.504, 95% CI = 1.131–2.001), and CSS (HR = 1.494, 95% CI = 1.109–2.012) (see Table 4). Reduced UA/Cr ratio level (≤3.98) was also a key predictive risk factor for OS (HR = 1.337, 95% CI = 1.004–1.780), DFS (HR = 1.376, 95% CI = 1.030–1.839), and CSS (HR = 1.420, 95% CI = 1.049–1.923) (see Table 5).

## 4. Discussion

The purpose of the present study, which involved 814 Chinese men with LSCC and 814 normal control subjects, was to evaluate the role of serum UA as an antioxidant molecule to protect against LSCC. It was found that serum UA levels were significantly lower among patients in the LSCC group compared to the sex- and age-matched control group without LSCC; in addition, the mean level of serum UA was lowest during stage IV, followed by stage III, stage II, and stage I. UA has been shown to be a biomarker for renal function; thus, UA/Cr ratios were used to reduce possible interference caused by differences in renal function [16, 29]. UA/Cr ratio levels in the LSCC group were lower than those in the control group, and the mean levels of the UA/Cr ratio were also lowest during stage IV, followed by stage III, stage II, and stage I. The multivariate Cox regression analysis showed that reduced UA levels (<0.315) and lower UA/Cr ratios (<3.98) were key predictive risk factors for OS, DFS, and CSS. These results weigh strongly against the idea that high UA concentrations reflect more serious prognoses [24, 25], suggesting instead that high serum UA concentrations may have a protective effect against LSCC because of their antioxidant properties.

From the above results, the following two questions arise: First, why is the role of serum UA controversial in patients with cancer? Second, why was the concentration of serum UA lower in patients with LSCC? Limited data are available in the literature regarding the association of serum UA levels with LSCC. Two theories about the function of serum UA in cancer exist: one is that UA has a protective effect on cancer due to its antioxidant properties [15] and the other is that the prooxidant role of UA induces proliferation and inflammation and is involved in intracellular redox-dependent mechanisms [30]. It is particularly interesting that UA plays this dual role as an antioxidant and prooxidant in patients with cancer. The precise relationship between serum UA and cancer remains obscure and therefore needs further elucidation.

In response to why serum UA concentrations are lower in patients with LSCC, high serum UA concentrations may have a protective effect against LSCC. Regarding this association, different factors might explain the prognostic value of serum UA: (1) a very obvious explanation could be that the patients with the most advanced disease (who therefore have swallowing problems) perhaps had changed their food intake to a lower purine-containing diet before their operations, (2) the lower levels are a result of decreased production and increased consumption of UA, and (3) UA secretion increases through the kidney [31]. It was first thought that it was impossible for the increase in UA excreted through the kidney to reduce UA levels for the following reasons: (1) the level of Cr, also excreted through the kidney, increases in patients with LSCC; (2) the UA/Cr ratio reduces the possible interference caused by differences in renal function (the UA/Cr ratio level in the LSCC group in the present study was lower than that in the control group); (3) the patients with renal dysfunction were excluded from this study; and (4) levels of UA were pretreatment, meaning the influence from surgery and medicine for UA was limited. This indicates that lower UA concentrations may result from decreased production and heightened consumption of UA. Thus, low serum UA levels can be regarded as a surrogate biomarker for poor tumour biology.

In addition to the role of UA as a simple biomarker, its independent prognostic role might also be explained by its antioxidant properties on tumour progression. UA is a well-known natural antioxidant present in fluids and tissues throughout the body and may contribute to up to two-thirds of the antioxidant capacity of human blood [32, 33]. Previous studies have indicated that oxidative stress plays an important role in tumour cell formation and reproduction [34]. The oxidative stress damage to tumour cells, reflected by a low UA level, results in a tumour microenvironment enriched with an inflammatory response. On the other hand, LSCC patients with swallowing problems may have changed their food intake to a lower purine-containing diet, which accelerated tumour progression. Thus, UA may represent not only a response to tumour prognosis but may also contribute to the opsonisation and elimination of tumour cells.

In the present study, decreased UA levels (≤0.315) and UA/Cr ratios (≤3.98) were found to be key predictive risk factors for OS, DFS, and CSS. When the OS, DFS, and CSS curves were analysed via the Kaplan-Meier method and the differences assessed using a log-rank test, it was found that the UA and UA/Cr ratio levels were all positively associated with the prognosis of patients with LSCC in terms of OS, DFS, and CSS. Therefore, elevated UA levels could indicate better prognoses, a finding supported by Dziaman et al.[18] who showed that elevated levels of UA corresponded with longer survival times among colorectal cancer patients. As an antioxidant, UA might be consumed by oxidizing agents to prevent an oxidative stress response. The level of reactive oxygen radicals would then increase due to the decrease in UA; low serum UA concentrations might be associated with the outcome to reflect more serious prognostic indications. This study proposes that lower levels of serum UA may contribute to antioxidant deficiency, which could accelerate LSCC development.

As this was the first study to focus on the evaluation of serum UA levels and UA/Cr ratios and their relationship with LSCC, it had some limitations. First, this was a single-center study, which limited the researchers' ability to explore the mechanisms underlying the associations between UA and LSCC. To compensate for this, further longitudinal studies are needed. Second, differences in dietary customs and their possible effects on serum UA concentration were not considered in the present study; thus, the UA results may not apply to populations outside of China. Only serum UA/Cr ratios were analysed with consideration for possible interference from diet customs, difference in the environment, and differences in renal function. Third, the weakness of this paper is the assumption that decreased serum UA results in increased cancer risk and not perhaps that more aggressive cancers result in decreases in uric acid levels. Fourth, the cancer cohort started with different pretreatment levels compared to the noncancer cohort, which may have introduced biases. Finally, education, liver function, total cholesterol, triglycerides, and income may be confounding factors, but we were not able to evaluate these factors in our study. Therefore, a forward-looking, multicenter study with a larger sample size should be conducted.

## 5. Conclusion

Our study provides evidence to indicate that pretreatment UA and UA/Cr ratios have significant associations with cancer progression, OS, DFS, and CSS and that these parameters could be considered independent prognostic values for patients with LSCC.

## Figures and Tables

**Table 1:** Baseline demographics and lifestyle characteristics of laryngeal squamous cell cancer patients.

Covariates	Number of patients/mean
No. of individuals	814
Age at diagnosis (years), mean, range	60.66, 27-89
>60	399
≥60	415
Body mass index (kg/m^2^), mean, range	22.94, 16.72-28.43
Male (%)	100%
Death events	191
Duration of follow-up (months), median, range	72, 3-116
Smoking history (yes/no)	570/244
Drinking history (yes/no)	309/505
Hypertension (yes/no)	229/585
Median recurrence (months), range	23, 1-95
T stage	
T_1_	195
T_2_	328
T_3_	239
T_4_	52
N stage	
N_0_	722
N_1_	31
N_2_	52
N_3_	9
TNM stage	
I	195
II	302
III	216
IV	101
Tumour size (cm), mean, range	1.80, 0-6.5
Tumour size (≤2 cm/>2 cm)	511/303
Neck dissection (yes/no)	145/669
Operation therapy	
Total laryngectomy	326
Partial laryngectomy	488
Tumour subsite	
Supraglottic	179
Subglottic	11
Glottic	624

**Table 2:** Comparison of UA and the UA/Cr ratio in patients with LSCC, stratified according to demographics and clinical characteristics.

	UA		UA/Cr ratio		Cr	
	Mean ± SD	*P*	Mean ± SD	*P*	Mean ± SD	*P*
TNM stage						
I (*n* = 195)	0.339 ± 0.076		4.00 ± 0.99		83.88 ± 15.13	
II (*n* = 302)	0.337 ± 0.072		4.16 ± 1.09		84.65 ± 16.88	
III (*n* = 216)	0.325 ± 0.075		4.01 ± 0.96		83.81 ± 26.99	
IV (*n* = 101)	0.302 ± 0.079	<0.05^b,c,d,e,f^	3.79 ± 1.03	<0.05^e^	81.08 ± 13.17	0.463
T stage						
T_1_ (*n* = 195)	0.339 ± 0.076		4.00 ± 0.99		83.88 ± 15.13	
T_2_ (*n* = 328)	0.337 ± 0.073		4.15 ± 1.08		84.79 ± 16.58	
T_3_ (*n* = 239)	0.323 ± 0.076		4.02 ± 0.99		82.96 ± 25.91	
T_4_ (*n* = 52)	0.283 ± 0.070	<0.05^b,c,d,e,f^	3.57 ± 0.99	<0.05^c,e,f^	81.08 ± 14.13	0.506
N stage						
N_0_ (*n* = 722)	0.331 ± 0.074		4.04 ± 1.03		84.13 ± 19.88	
N_1_ (*n* = 31)	0.316 ± 0.080		3.94 ± 0.95		82.03 ± 16.98	
N_2_ (*n* = 52)	0.314 ± 0.077		3.96 ± 0.96		80.44 ± 13.67	
N_3_ (*n* = 9)	0.374 ± 0.106	<0.05^b,d^	4.64 ± 1.44	0.313	82.44 ± 11.97	0.692
Age						
<60 (*n* = 399)	0.336 ± 0.075		4.18 ± 1.04		82.14 ± 13.90	
≥60 (*n* = 415)	0.325 ± 0.076	0.033	3.90 ± 1.02	<0.001	85.40 ± 23.37	0.015
BMI						
<22.94 (413)	0.329 ± 0.078		4.00 ± 1.02		84.93 ± 23.83	
≥22.94 (401)	0.331 ± 0.074	0.685	4.06 ± 1.04	0.466	83.21 ± 16.54	0.229
Hypertension						
No (*n* = 585)	0.324 ± 0.072		4.00 ± 1.03		82.58 ± 14.59	
Yes (*n* = 229)	0.347 ± 0.082	<0.001	4.14 ± 1.06	0.087	86.92 ± 27.92	0.026
Smoking						
No (*n* = 244)	0.331 ± 0.073		4.06 ± 1.05		83.30 ± 15.50	
Yes (*n* = 570)	0.330 ± 0.076	0.749	4.03 ± 1.03	0.636	84.01 ± 20.82	0.633
Drinking						
No (*n* = 505)	0.327 ± 0.073		3.96 ± 0.98		83.96 ± 16.95	
Yes (*n* = 309)	0.336 ± 0.078	0.107	4.16 ± 1.11	0.010	83.53 ± 22.82	0.757
Tumour size						
≤2 cm (*n* = 511)	0.337 ± 0.072		84.28 ± 16.54		4.07 ± 1.02	
>2 cm (*n* = 303)	0.318 ± 0.080	0.001	82.89 ± 23.87	0.332	3.97 ± 1.07	0.183
Operation therapy						
Partial laryngectomy (*n* = 488)	0.344 ± 0.072		85.92 ± 18.16		4.03 ± 0.98	
Total laryngectomy (*n* = 326)	0.318 ± 0.076	<0.001	83.48 ± 23.53	0.213	3.96 ± 1.05	0.450
Neck dissection						
No (*n* = 669)	0.333 ± 0.075		4.03 ± 1.03		84.61 ± 20.05	
Yes (*n* = 145)	0.324 ± 0.073	0.262	4.09 ± 0.97	0.557	81.02 ± 15.58	0.070
Tumour subsite						
Supraglottic (*n* = 179)	0.318 ± 0.076		4.08 ± 1.09		80.37 ± 17.36	
Glottis (*n* = 624)	0.334 ± 0.074	0.013	4.03 ± 1.01	0.601	84.79 ± 19.89	0.007
Subglottic (*n* = 11)	0.298 ± 0.095	—	3.68 ± 1.35	—	83.45 ± 15.32	—

Data are expressed as mean ± standard deviation (SD). The chi-square test and one-way ANOVA were used. UA: uric acid; Cr: creatinine; BMI: body mass index. ^a^ *P* < 0.05 for the difference between stage I and stage II (1-way ANOVA with the LSD post hoc test). ^b^ *P* < 0.05 for the difference between stage I and stage III (1-way ANOVA with the LSD post hoc test). ^c^ *P* < 0.05 for the difference between stage I and stage IV (1-way ANOVA with the LSD post hoc test). ^d^ *P* < 0.05 for the difference between stage II and stage III (1-way ANOVA with the LSD post hoc test). ^e^ *P* < 0.05 for the difference between stage II and stage IV (1-way ANOVA with the LSD post hoc test). ^f^ *P* < 0.05 for the difference between stage III and stage IV (1-way ANOVA with the LSD post hoc test).

**Table 3:** Multiple linear regressions for associations between UA, Cr, and the UA/Cr ratio with severity (TNM) of LSCC.

Linear regression	*B*	*P* value	95% CI
Cr	-0.003	0.237	-0.013-0.003
UA	-1.824	<0.001	-2.437-1.002
UA/Cr ratio	-0.079	0.045	-0.321-0.024

UA: uric acid; Cr: creatinine. Adjusted for age, body mass index, hypertension, smoking, and drinking.

**Table 4:** Univariate Cox regression analysis for overall survival, disease-free survival, and cancer-specific survival in patients with LSCC.

	OS		DFS		CSS	
	HR (95% CI)	*P*	HR (95% CI)	*P*	HR (95% CI)	*P*
UA						
UA > 0.315	1		1		1	
UA ≤ 0.315	1.466 (1.104-1.947)	0.008	1.463 (1.101-1.943)	0.009	1.457 (1.084-1.959)	0.013
UA/Cr ratio						
UA/Cr ratio > 3.98	1		1		1	
UA/Cr ratio ≤ 3.98	1.308 (0.984-1.737)	0.044	1.350 (1.011-1.802)	0.042	1.391 (1.028-1.881)	0.032
TNM stage						
I-II (*n* = 497)	1		1		1	
III-IV (*n* = 317)	2.714 (2.034-3.620)	<0.001	2.882 (2.160-3.845)	<0.001	3.106 (2.291-4.211)	<0.001
T stage						
T_1-2_ (*n* = 523)	1		1		1	
T_3-4_ (*n* = 291)	2.391 (1.799-3.176)	<0.001	2.532 (1.905-3.365)	<0.001	2.726 (2.025-3.671)	<0.001
N stage						
N_0_ (*n* = 722)						
N_1_ (*n* = 31)	1		1		1	
N_2-3_ (*n* = 61)	3.254 (2.123-4.783)	<0.001	3.409 (2.319-5.012)	<0.001	3.434 (2.317-5.090)	<0.001
Tumour subsite						
Glottic and subglottic (*n* = 635)	1		1		1	
Supraglottic (*n* = 179)	2.246 (1.675-3.013)	<0.001	2.382 (1.776-3.196)	<0.001	2.128 (1.653-2.975)	<0.001
Operation therapy						
Partial laryngectomy (*n* = 488)	1		1		1	
Total laryngectomy (*n* = 326)	2.473 (1.880-3.253)	<0.001	2.621 (1.992-3.449)	<0.001	2.468 (1.876-3.247)	<0.001
Tumour size						
≤2 cm (*n* = 511)	1		1		1	
>2 cm (*n* = 303)	2.650 (1.994-3.523)	<0.001	2.633 (1.981-3.500)	<0.001	2.766 (2.081-3.678)	<0.001
Age						
<60 (*n* = 399)	1		1		1	
≥60 (*n* = 415)	1.678 (1.253-2.247)	0.001	1.598 (1.193-2.139)	0.002	1.569 (1.160-2.122)	0.003
BMI						
<22.94 (413)	1		1		1	
≥22.94 (401)	0.841 (0.628-1.127)	0.246	0.855 (0.638-1.144)	0.292	0.859 (0.638-1.157)	0.316
Hypertension						
No (*n* = 585)	1		1		1	
Yes (*n* = 229)	1.254 (0.926-1.698)	0.143	1.300 (0.960-1.760)	0.090	1.218 (0.887-1.672)	0.224
Smoking history						
No (*n* = 244)	1		1		1	
Yes (*n* = 570)	1.057 (0.773-1.446)	0.727	1.103 (0.806-1.509)	0.540	1.106 (0.795-1.538)	0.549
Drinking history						
No (*n* = 505)	1		1		1	
Yes (*n* = 309)	1.111 (0.833-1.483)	0.474	1.162 (0.871-1.551)	0.308	1.139 (0.844-1.537)	0.396

UA: uric acid; UA/Cr ratio: uric acid/creatinine ratio; BMI: body mass index.

**Table 5:** Multivariate Cox regression analysis for overall survival, disease-free survival, and cancer-specific survival in patients with LSCC.

	OS		DFS		CSS	
	HR (95% CI)	*P*	HR (95% CI)	*P*	HR (95% CI)	*P*
UA						
UA > 0.315	1		1		1	
UA ≤ 0.315	1.458 (1.095-1.942)	0.010	1.504 (1.131-2.001)	0.005	1.494 (1.109-2.012)	0.008
UA/Cr ratio						
UA/Cr ratio > 3.98	1		1		1	
UA/Cr ratio ≤ 3.98	1.337 (1.004-1.780)	0.047	1.376 (1.030-1.839)	0.031	1.420 (1.049-1.923)	0.023
TNM stage						
I-II (*n* = 497)	1		1		1	
III-IV (*n* = 317)	2.732 (2.041-3.658)	<0.001	2.871 (2.145-3.842)	<0.001	3.131 (2.301-4.260)	<0.001
T stage						
T_1-2_ (*n* = 523)	1		1		1	
T_3-4_ (*n* = 291)	2.404 (1.804-3.202)	<0.001	2.524 (1.896-3.361)	<0.001	2.738 (2.028-3.697)	<0.001
N stage						
N_0_ (*n* = 722)						
N_1_ (*n* = 31)	1		1		1	
N_2-3_ (*n* = 61)	3.196 (2.172-4.702)	<0.001	3.136 (2.253-4.882)	<0.001	3.372 (2.273-5.004)	<0.001
Tumour subsite						
Glottic and subglottic (*n* = 635)	1		1		1	
Supraglottic (*n* = 179)	2.205 (1.641-2.963)	<0.001	2.179 (1.621-2.927)	<0.001	2.332 (1.737-3.132)	<0.001
Operation therapy						
Partial laryngectomy (*n* = 488)	1		1		1	
Total laryngectomy (*n* = 326)	2.371 (1.794-3.135)	<0.001	2.508 (1.897-3.316)	<0.001	2.368 (1.791-3.130)	<0.001
Tumour size						
≤2 cm (*n* = 511)	1		1		1	
>2 cm (*n* = 303)	2.640 (1.981-3.517)	<0.001	2.624 (1.969-3.497)	<0.001	2.724 (2.045-3.627)	<0.001
Body mass index						
<22.94 (413)	1		1		1	
≥22.94 (401)	0.845 (0.631-1.132)	0.259	0.842 (0.629-1.129)	0.250	0.854 (0.637-1.144)	0.289
Age						
<60 (*n* = 399)	1		1		1	
≥60 (*n* = 415)	1.674 (1.241-2.259)	0.001	1.585 (1.175-2.139)	0.003	1.577 (1.157-2.149)	0.004
Hypertension						
No (*n* = 585)	1		1		1	
Yes (*n* = 229)	1.142 (0.839-1.554)	0.397	1.137 (0.836-1.548)	0.414	1.193 (0.877-1.624)	0.262
Smoking history						
No (*n* = 244)	1		1		1	
Yes (*n* = 570)	1.075 (0.752-1.536)	0.692	1.082 (0.757-1.545)	0.666	1.095 (0.766-1.567)	0.618
Drinking history						
No (*n* = 505)	1		1		1	
Yes (*n* = 309)	1.132 (0.816-1.570)	0.459	1.129 (0.814-1.566)	0.467	1.162 (0.837-1.614)	0.369

UA: uric acid; UA/Cr ratio: uric acid/creatinine ratio.

## Data Availability

The data used to support the findings of this study are available from the corresponding authors upon request.

## Supplementary Materials

Supplementary Materials Table S1: comparison of UA, the UA/Cr ratio, and Cr between the LSCC group and the control group. Table S2: comparison of demographics and clinical characteristics in patients with LSCC, stratified according to UA and the UA/Cr ratio. Table S3: the number of subjects in different groups, according to UA and the UA/Cr ratio.
