# Supplementary material for: Pretreatment Serum Uric Acid as an Efficient Predictor of Prognosis in Men with Laryngeal Squamous Cell Cancer: A Retrospective Cohort Study
Source: Oxid Med Cell Longev. 2019 Apr 16;2019:1821969. doi: 10.1155/2019/1821969 (PMC6501142; doi:10.1155/2019/1821969)
Supplement: Supplementary Materials — Table S1: comparison of UA, the UA/Cr ratio, and Cr between the LSCC group and the control group. Table S2: comparison of demographics and clinical characteristics in patients with LSCC, stratified according to UA and the UA/Cr ratio. Table S3: the number of subjects in different groups, according to UA and the UA/Cr ratio. [file 1821969.f1.pdf]

Pre-treatment serum uric acid as an efficient predictor of prognosis in men with laryngeal squamous cell cancer: a retrospective cohort study

Chiyao Hsueh[1,2], Mingxi Shao[3], Wenjun Cao[3], Shengjie Li[3*],Liang Zhou[1, 2*]

1. Department of Otolaryngology, Eye & ENT Hospital, Shanghai Medical College, Fudan University, Shanghai, China.

2. Shanghai Key Clinical Disciplines of Otorhinolaryngology, Shanghai Medical College, Fudan University, Shanghai, China.

3. Department of Clinical Laboratory, Eye & ENT Hospital, Shanghai Medical College, Fudan University, Shanghai, China

Table S1. Comparison of UA, UA/Cr ratio, and Cr between LSCC group and control group

	LSCC group	Control group	t value	P value
Age (years)	60.66±9.81	60.66±9.81	0.000	1.000
Gender (male)	814	814		
BMI (kg/m^2)	22.94±3.22	22.86±3.10	0.431	0.667
UA (mmol/l)	0.330±0.075	0.356±0.078	6.850	<0.001
Cr (umol/l)	83.80±19.37	79.08±18.10	5.083	<0.001
UA/Cr ratio	4.04±1.04	4.62±1.12	10.847	<0.001
Logistic regression	OR	P value	95%CI	
Cr	1.003	0.664	0.989-1.017	
UA	0.671	0.002	0.018-2.165	
UA/Cr ratio	0.584	<0.001	0.439-0.778	

Data are expressed as mean ± standard deviation (SD). Independent student's t-test and Logistic regression analyze was used. UA: uric acid. Cr: creatinine, BMI: body mass index.

Table S2. Comparison of demographics and clinical characteristic in patients with LSCC, stratified according to UA and UA/Cr ratio

	UA			UA/Cr ratio		
	UA >0.315	UA ≤0.315	P	UA/Cr ratio >3.98	UA/Cr ratio ≤3.98	P
TNM stage						
Ⅰ (n=195)	104	91		87	108	
Ⅱ (n=302)	183	119		156	146	
Ⅲ(n=216)	121	95		52	164	
Ⅳ(n=101)	33	68	<0.001	37	64	<0.001
T stage						
T_1 (n=195)	104	91		87	108	
T_2 (n=328)	199	129		168	160	
T_3(n=239)	114	115		113	126	
T_4(n =52)	14	38	<0.001	14	38	0.011
N stage						
N_0 (n=722)	453	269		341	381	
N_1 (n=31)	16	15		13	18	
N_2 (n=52)	22	30		25	27	
N_3 (n=9)	4	5	0.012	3	6	0.801
Age						
<60 (n=399)	243	156		188	211	
≥60(n=415)	198	217	0.087	194	221	0.916
BMI						
<22.94 (413)	216	197		189	224	
≥22.94 (401)	225	176	0.275	193	208	0.499
Hypertension						
No (n=585)	335	253		268	317	
Yes (n=229)	109	120	0.016	114	115	0.308
Smoking history						
No (n=244)	148	96		112	132	
Yes (n=570)	293	277	0.015	270	300	0.701
Drinking history						
No (n=505)	290	215		227	278	
Yes (n=309)	151	158	0.017	155	154	0.148
Tumor subsite						
Supraglottic (n=179)	89	90		85	94	
Glottis(n=624)	348	276		297	327	
Subglottic(n=11)	4	7	0.176	3	8	0.408

UA: uric acid. UA/Cr ratio: uric acid/creatinine, BMI: body mass index.

Table S3. The number of subjects in different group, according to UA and UA/Cr ratio

	Ⅰ stage	Ⅱ stage	Ⅲ stage	Ⅳ stage	LSCC group	Control group	P value
UA							
UA >0.315	104	183	121	33	441	519	
UA≤0.315	91	119	95	68	373	206	<0.05[a], 0.006[b]
UA/Cr ratio							
UA/Cr ratio ≤3.98	108	146	164	64	432	202	
UA/Cr ratio >3.98	87	156	52	37	382	523	<0.001[a],<0.001[b]

Chi-square test was used.

aP<0.05 for the difference between LSCC group and Control group.

bP-trender <0.05 for the trender among Ⅰ stage, Ⅱ stage, Ⅲ stage, Ⅳ stage.
